# Newly described anatomical opening on forelimb tendon in the artiodactyls and its relation to knee clicks

**DOI:** 10.1038/s41598-022-08303-z

**Published:** 2022-03-14

**Authors:** Martin Pyszko, Petr Němeček, Ondřej Horák, Václav Páral, Radim Kotrba, Louwrens C. Hoffman, Jan Robovský

**Affiliations:** 1 Department of Anatomy, Histology & Embryology, Faculty of Veterinary Medicine, University of Veterinary Sciences Brno, Palackého třída 1946/1, 612 42 Brno, Czech Republic; 2 Jiří Orten Grammar School, Jaselská 932, 284 80 Kutná Hora, Czech Republic; 3 Department of Ethology, Institute of Animal Science, 104 00 Prague 10 - Uhříněves, Czech Republic; 4 Department of Animal Science and Food Processing, Faculty of Tropical AgriSciences, Czech University of Life Sciences Prague, Kamýcká 129, 165 00 Praha 6 - Suchdol, Czech Republic; 5 Department of Animal Sciences, University of Stellenbosch, Matieland, Private Bag X1, Stellenbosch, 7602 South Africa; 6 Centre for Nutrition and Food Sciences, Queensland Alliance for Agriculture and Food Innovation (QAAFI), The University of Queensland, Digital Agricultural Building, 8115, Office 110, Gatton, 4343 Australia; 7 Department of Zoology, Faculty of Science, University of South Bohemia, Branišovská 1760, 370 05 České Budějovice, Czech Republic; 8 Liberec Zoo, Lidové sady 425/1, 460 01 Liberec, Czech Republic

**Keywords:** Zoology, Biomechanics

## Abstract

To understand which morphological/anatomical parts may be responsible in artiodactyl ungulates for the clicking sound made when moving, this research focuses on the forelimb tendon apparatus where an undescribed opening in the fibrous cuff (*manica flexoria*), called hereafter for its shape as an “oval window” in the *manica flexoria* (OWMF), was detected. This oval window was found in 24 of the 25 species of four families (Camelidae, Giraffidae, Cervidae, and Bovidae) evaluated; the exception being in *Bos taurus taurus* (Domestic cattle). The length and width of the OWMF enabled correct species discrimination between the majority of species, but remained conservative intraspecifically, as it did not differ between the left and right side of the forelimb, third and fourth digits, or between sexes. When evaluating the shape of OWMF in individual species, and measuring its length and width, 18 out of the 24 species investigated had this window as an oval shape, the remaining 25% of species exhibited more oval-oblong shapes with either proximal or distal asymmetry. The function of the OWMF in the thoracic autopodium of most ruminant even-toed ungulates is not yet fully understood. Its most likely function is to help balance the pressure inside the ligament cuff and reduce the friction of the touching surfaces of the muscle tendons—thus facilitating the movement of the digits when walking. None of the absolute or relative OWMF parameters fit exclusively with the occurrence and distribution of knee-clicks produced by some bovids and cervids during movement, so the mechanism responsible for this sound remains cryptic from the present anatomical perspective.

## Introduction

Some ungulates such as the Common eland (*Taurotragus oryx*; the taxonomy in the present paper follows Grubb^1^ due to its fitting with our inspected taxa) emit a clicking sound during walking^2^ which was explained as a signalling of male quality^3^ and as part of an explanation of the multimodality within the signal^4^. This phenomenon has only been investigated in detail in the Common eland by the above-mentioned studies; as only males emit knee-clicks in this species. Knee-clicks are described in several other ungulate species^5–10^. Authors of this manuscript have documented such sounds in additional species, for example, in some Caprinae (JR in preparation), but these clicks are regularly and loudly emitted by only the following species according to our observations: Père David's deer (*Elaphurus davidianus*), White-lipped deer (*Przewalskium albirostris*), Reindeer (*Rangifer tarandus*), and Common eland (*Taurotragus oryx*)—in the first three species knee-clicks are emitted by both sexes. The function of the clicking and how this sound is produced, is unknown. The clicks are emitted during walking and running, and when the individual changes weight on its legs^5,11^. The majority of publications agree that it is emitted from the thoracic autopod, but no agreement exist from where exactly^2,3,6,7,11,12^. Since some authors postulate that the click is produced when a tendon slips over a carpal bone^2,3^, the complete tendon apparatus of the forelimb in even-toed ungulates (Artiodactyla) in respect of knee-clicking was inspected in this investigation.

Artiodactyl legs are very effective organs which have been transformed and optimized by species through decades of selection for occupying/surviving extremely diverse habitats, decreasing transport costs and escaping from predation^13–20^. Currently, it has been proven as a reliable environmental predictor of ecoregion, vegetation cover and precipitation worldwide^21^. Every transformation has required a complex integrative adaptation of diverse tissues, including that by the tendons^14,16^.

Tendons in the thoracis autopod are configured into a fibrous cuff, called the *manica flexoria,* which in ruminant even-toed ungulates consists of distal sections of the *flexor digitorum superficialis* and *adductor digiti II* and *V*^22,23^. The tendon of the deep digital flexor (*flexor digitorum profundus*) runs through the interior of this "cuff", which takes the form of two ligament tubes. *Manica flexoria* is located on the palmar surface of the so-called metacarpophalangeal joint and its function is to fix the tendons of the digital flexor near the bone base^24,25^. Three muscles are associated with this thoracious autopod, specifically the *musculus flexor digitorum superficialis*, the *musculus flexor digitorum profundus*, and the *musculi adductores digitorum* (for details see Appendix S1). The distribution of *manica flexoria* and the mentioned muscles varies in modern ungulates which probably indicates either some shared evolutionary transitions or an independent origin of some structures (see below in “Discussion”).

Besides knowledge about the diversity, distribution, and evolutionary significance of these structures, the knowledge of the anatomical structure of the thoracic autopod also has practical significance. For the correct interpretation of the results, it is recommended that various diagnostic and imaging methods typically used in veterinary medicine be utilised. These are, for example, endoscopic examination (tenoscopy), X-rays (radiology), USG (ultrasonography), CT (computed tomography), and MRI (magnetic resonance imaging). Nogueira et al.^26^, and Bertagnoli et al.^27^, supplemented USG by an endoscopic study of the common tendon sheath of bovine digital flexors and structures located in its vicinity. Ultrasonographic diagnosis of soft tissues at the distal end of cattle limbs were also performed by Kofler and Edinger^28^ whilst Blaser et al.^29^ dealt with arthroscopy of the bovine spinal joint and surrounding structures. The above-mentioned imaging methods have also been used in other domestic species. Of the various publications, it is worth mentioning, for example, a study on endoscopy of the *fibrous vagina* of a digital flexor in a horse^30^ as well as ultrasonography of many structures of the horse's forelimb^31–33^. El-Shafey and Kassab^34^ compared CT with transverse sections of metatarsus and digits in the One-humped camel (*Camelus dromedarius*) and the Water buffalo (*Bubalus bubalis*).

As only a weak or no clear indication exists in the literature of the anatomical part of the autopod responsible for the clicking sound as described for several ungulates, comparative dissections of various wild and domestic even-toed ungulates were made to gain more insight on this phenomenon. As the knee-clicking sound is emitted more regularly and loudly in males, males and females were also compared so as to identify whether males have a different anatomy of the autopod. So as to determine whether these anatomical differences develop during ontogeny or whether ungulates are born with identical anatomy in comparison to adults, calves and/or juveniles were included in the investigations.

## Results

In the pilot trial phase, identification of the source of the knee-clicks sounds through the use of an acoustic camera on a live animal (tame adult eland bull) yielded inconclusive results. The acoustic camera did not highlight a single area on the forelimb during sound emission as a possible source of the click sounds. The sound recordings were contaminated by the sounds reflecting off installations such as the pen walls around the animal as well as by other sounds eminating from the surroundings. Therefore, a biomechanical approach post-mortem via different limb positions and pressure involved on different parts of the limbs to mimic movement of the limb during walking was utilized. This was conducted on the whole limb of an adult eland antelope, but no sound or vibrations were detected on the limb 24 h post-mortem; probably because it was not possible to simulate the movement and loading of the limb properly after 24 h. Based on the above, it was decided to focus on an anatomical approach in an attempt to identify the source of the knee-clicks.

In general, the anatomy of the autopodium exhibited a significant conservatism across the analysed species of the three ruminant families, thereby indicating some evolutionary and/or functional constraints. The *manica flexoria* of both camelids exhibited distinctly different patterns due to the lack of the *musculi adductores digitorum*, the ligament tube is thus formed only by the tendon of the surface digital flexor supplemented by an auxiliary ligament strip, similar to that of a horse (Supplementary Fig. S1). However, this auxiliary ligament plate does not have as sharp and massive boundaries as the digital adduct tendon.

Besides this modification of the *manica flexoria* in the camelids, the only diverse structure observed were the oval windows on the adduct tendon facing the bone, which was named as an oval window in the *manica flexoria* (abbreviated as OWMF). The OWMF were observed in all species of Camelidae and Ruminantia, except in taurine domestic cattle. In this species, the OWMF was not found on either digit of both forelegs. This finding was the same for males and females and for all recognized age categories.

Dimensions of the OWMF, as well as their ratios, are summarized in Table 1. Briefly, the length of the OWMF ranged from 1 to 6 cm, with the shortest OWMF being observed in the Domestic goat and European mouflon: with the longest in the Bactrian camel and the Guanaco. The width of the OWMF ranged from 0.5 to 2 cm. The narrowest widths were recorded in the European mouflon and European bison, the widest in the Bactrian camel, and the Giraffe. The ratio of lengths to widths ranged from 1.4:1 for the Reindeer to 6.9:1 for the Guanaco. For the range and species with the smallest and largest ratios in relative scale see Table 1.

**Table 1:** Average dimensions (± standard deviation) of the oval opening in the *manica flexoria* (OWMF) and their ratios (using M + F values) in the inspected species.

Scientific name	Length (mm)	Length (mm)	Length (mm)	Width (mm)	Width (mm)	Width (mm)	Length/width ratio	Length/weight ratio*100	Width/weight ratio*100	Length–width/weight ratio*100
	M	F	M + F	M	F	M + F				
*Aepyceros melampus*	12.68 ± 0.12	12.09 ± 0.16	12.48 ± 0.31	5.17 ± 0.13	4.9 ± 0.12	5.08 ± 0.18	2.45	24.83	10.13	4.87
*Antidorcas marsupialis*	12.93 ± 0.10	12.44 ± 0.15	12.68 ± 0.27	5.14 ± 0.14	4.61 ± 0.11	4.88 ± 0.30	2.59	33.33	12.86	6.80
*Bison bonasus*	14.88 ± 0.04	x	14.88 ± 0.04	4.78 ± 0.04	x	4.78 ± 0.04	3.10	2.61	0.84	0.54
*Bos taurus taurus*	0	0	0	0	0	0	0	0	0	0
*Camelus bactrianus bactrianus*	x	60 ± 0.61	60 ± 0.61	x	18.04 ± 0.12	18 ± 0.14	3.33	11.43	3.43	0.63
*Capra hircus hircus*	10.36 ± 0.10	10.01 ± 0.13	10.19 ± 0.21	6.44 ± 0.09	6.11 ± 0.09	6.28 ± 0.19	1.62	25.50	15.75	4.05
*Capreolus capreolus*	12.4 ± 0.12	12.24 ± 0.13	12.32 ± 0.15	5.09 ± 0.11	4.73 ± 0.16	4.91 ± 0.22	2.51	51.68	20.59	10.55
*Cervus elaphus*	15.13 ± 0.11	14.90 ± 0.10	14.98 ± 0.15	7.05 ± 0.11	6.96 ± 0.11	6.99 ± 0.12	2.14	9.22	4.30	1.32
*Cervus nippon pseudaxis*	14.66 ± 0.15	14.13 ± 0.12	14.39 ± 0.30	4.86 ± 0.13	4.50 ± 0.07	4.68 ± 0.21	3.06	20.57	6.71	4.38
*Connochaetes gnou*	35.63 ± 0.26	35.00 ± 0.16	35.32 ± 0.38	10.40 ± 0.11	10.04 ± 0.14	10.22 ± 0.22	3.46	23.40	6.76	2.29
*Connochaetes taurinus taurinus*	20.15 ± 0.16	19.71 ± 0.12	19.93 ± 0.26	10.13 ± 0.08	9.94 ± 0.11	10.04 ± 0.14	1.99	9.47	4.76	0.95
*Damaliscus pygargus phillipsi*	20.08 ± 0.16	19.59 ± 0.10	19.84 ± 0.28	8.04 ± 0.10	7.50 ± 0.10	7.77 ± 0.29	2.54	30.46	12.00	3.91
*Elaphurus davidianus*	11.86 ± 0.09	11.63 ± 0.10	11.74 ± 0.15	5.91 ± 0.14	5.65 ± 0.09	5.78 ± 0.17	2.02	6.55	3.25	1.13
*Giraffa camelopardalis*	42.20 ± 0.22	41.65 ± 0.13	41.93 ± 0.33	14.08 ± 0.15	13.59 ± 0.13	13.84 ± 0.28	3.04	4.18	1.38	0.30
*Kobus megaceros*	19.98 ± 0.15	x	19.98 ± 8.98	8.98 ± 0.11	x	8.98 ± 0.11	2.22	22.22	10.00	2.47
*Lama glama guanicoe*	45.80 ± 0.19	x	45.80 ± 0.19	6.60 ± 0.12	x	6.60 ± 0.12	6.94	43.93	6.33	6.66
*Oryx beisa beisa*	20.94 ± 0.09	20.71 ± 0.09	20.83 ± 0.14	9.06 ± 0.09	8.70 ± 0.12	8.88 ± 0.21	2.34	12.30	5.26	1.38
*Oryx gazella*	12.04 ± 0.13	11.64 ± 0.13	11.84 ± 0.24	7.71 ± 0.11	7.37 ± 0.11	7.54 ± 0.20	1.57	6.85	4.36	0.91
*Ovis aries aries*	13.40 ± 0.10	13.05 ± 0.11	13.23 ± 0.20	7.91 ± 0.14	7.51 ± 0.13	7.71 ± 0.24	1.71	23.40	13.65	3.04
*Ovis aries musimon*	10.94 ± 0.15	10.74 ± 0.09	10.80 ± 0.15	4.90 ± 0.11	4.58 ± 0.11	4.69 ± 0.19	2.30	27.87	12.13	5.93
*Przewalskium albirostris*	12.71 ± 0.12	12.48 ± 0.13	12.59 ± 0.17	50.06 ± 0.09	4.78 ± 0.10	4.92 ± 0.17	2.57	7.65	2.98	1.56
*Rangifer tarandus*	11.78 ± 0.08	11.40 ± 0.07	11.53 ± 0.19	8.50 ± 0.07	8.24 ± 0.13	8.33 ± 0.17	1.39	9.97	7.19	1.20
*Taurotragus oryx*	13.58 ± 0.12	13.25 ± 0.10	13.42 ± 0.20	7.48 ± 0.11	7.02 ± 0.32	7.25 ± 0.34	1.86	2.52	1.35	0.35
*Tragelaphus spekii gratus*	12.10 ± 0.07	x	12.10 ± 0.07	7.80 ± 0.07	x	7.80 ± 0.07	1.55	14.24	9.18	1.83
*Tragelaphus strepsiceros*	20.88 ± 0.13	19.88 ± 0.12	20.08 ± 0.24	10.04 ± 0.12	9.75 ± 0.11	9.90 ± 0.19	2.03	10.05	4.95	1.02

*F* female, *M* male, *x* not available.

**Figure 1:**
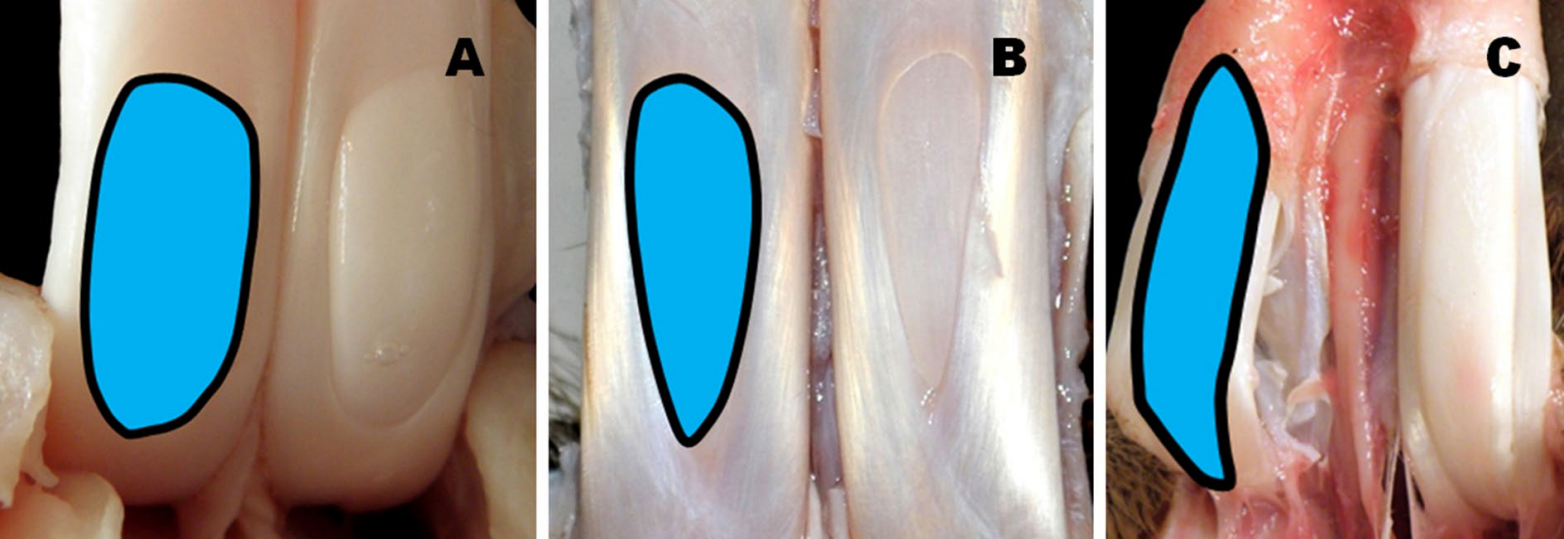
Variable shapes of the “oval window” in the *manica flexoria* of the pectoral limb (view of the adductor area). (**A**) European roe deer, (**B**) Indochinese sika deer, (**C**) Guanaco. Photos by M. P.

**Figure 2:**
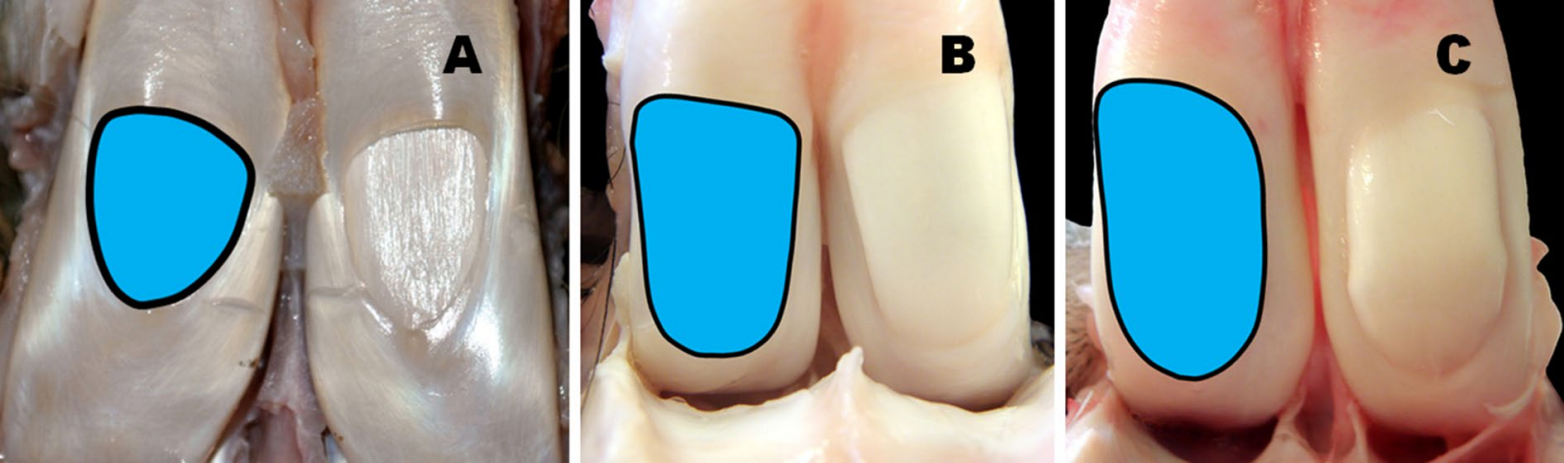
Variable shapes of the “oval window” in the *manica flexoria* of the pectoral limb (view of the adductor area). (**A**) Reindeer, (**B**) Domestic sheep, (**C**) Beisa oryx. Photos by M. P.

**Figure 3:**
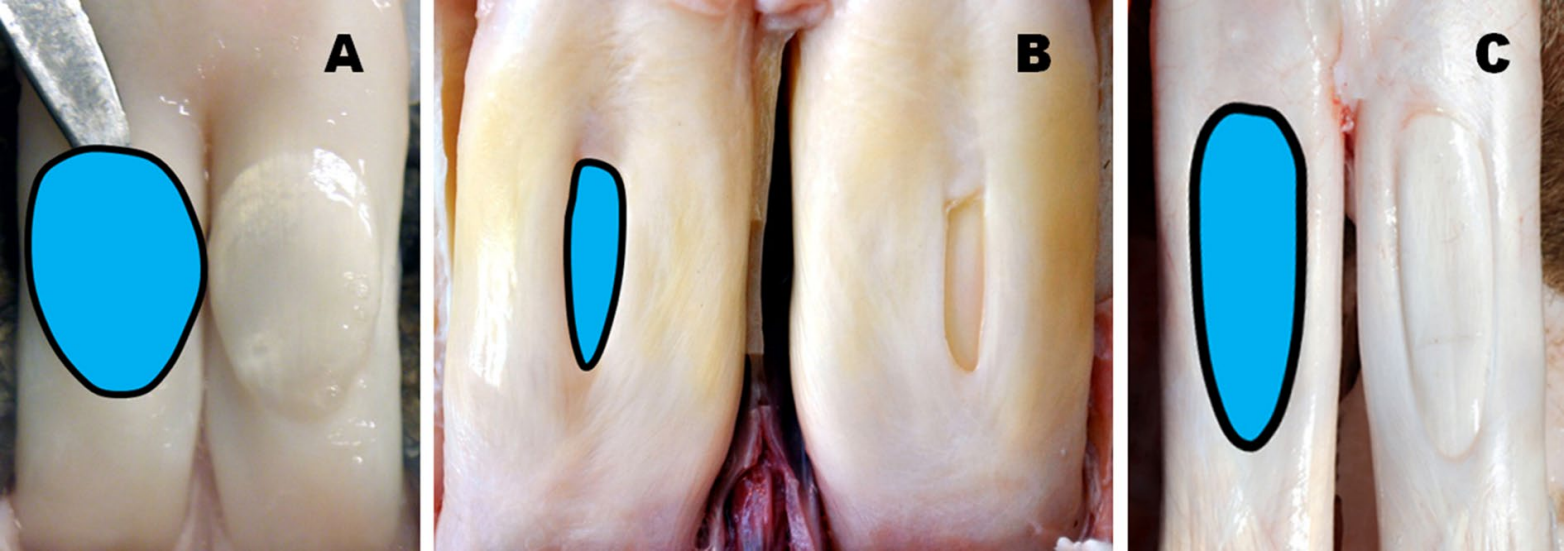
Variable shapes of the “oval window” in the *manica flexoria* of the pectoral limb (view of the adductor area). (**A**) Western sitatunga, (**B**) European bison, (**C**) Giraffe. Photos by M. P.

When evaluating the shape of the OWMF (Figs. 1, 2, 3) and measuring its length and width, 18 of the 24 species in the study (i.e., 75%) had this oval window (Fig. 1A) with a length to width ratio of 1.75–3.5:1. The remaining 25% (i.e., 6 species of even-toed ungulates) displayed oval-elongated shaped OWMF with proximal or distal asymmetry. For these species, the OWMF had the appearance of a "drop" (Fig. 3B), an "inverted drop" (Figs. 1B, 3C), a "rectangle" (Fig. 2B,C), a "triangle" (Fig. 2A), a "spindle" (Fig. 1C) or an "ovoid" shape (Fig. 3A).

In overview, four basic OWMF groups according to the shape and mutual ratio of its length and width were distinguished:

1. Oval shortened shape—length to width OWMF ratio 1.25–1.75:1—in Domestic goat, Domestic sheep (Fig. 2B), Gemsbok, Reindeer (Fig. 2A), and Western sitatunga (Fig. 3A);
2. Oval shape—length to width OWMF ratio 1.75–2.25:1—in Blue wildebeest, Common eland, Greater kudu, Nile lechwe, Père David's deer (Supplementary Fig. S4A), and Red deer;
3. Oval elongated shape—length to width OWMF ratio 2.25–2.75:1—in Beisa oryx (Fig. 2C), Blesbok, European mouflon, European roe deer (Fig. 1A), Impala, Springbok, and White-lipped deer;
4. Oval shape very elongated—length to width OWMF ratio more than 2.75:1—in Bactrian camel, Black wildebeest, European bison (Fig. 3B), Giraffe (Fig. 3C), Guanaco (Fig. 1C), and Indochinese sika deer (Fig. 1B).

**Figure 4:**
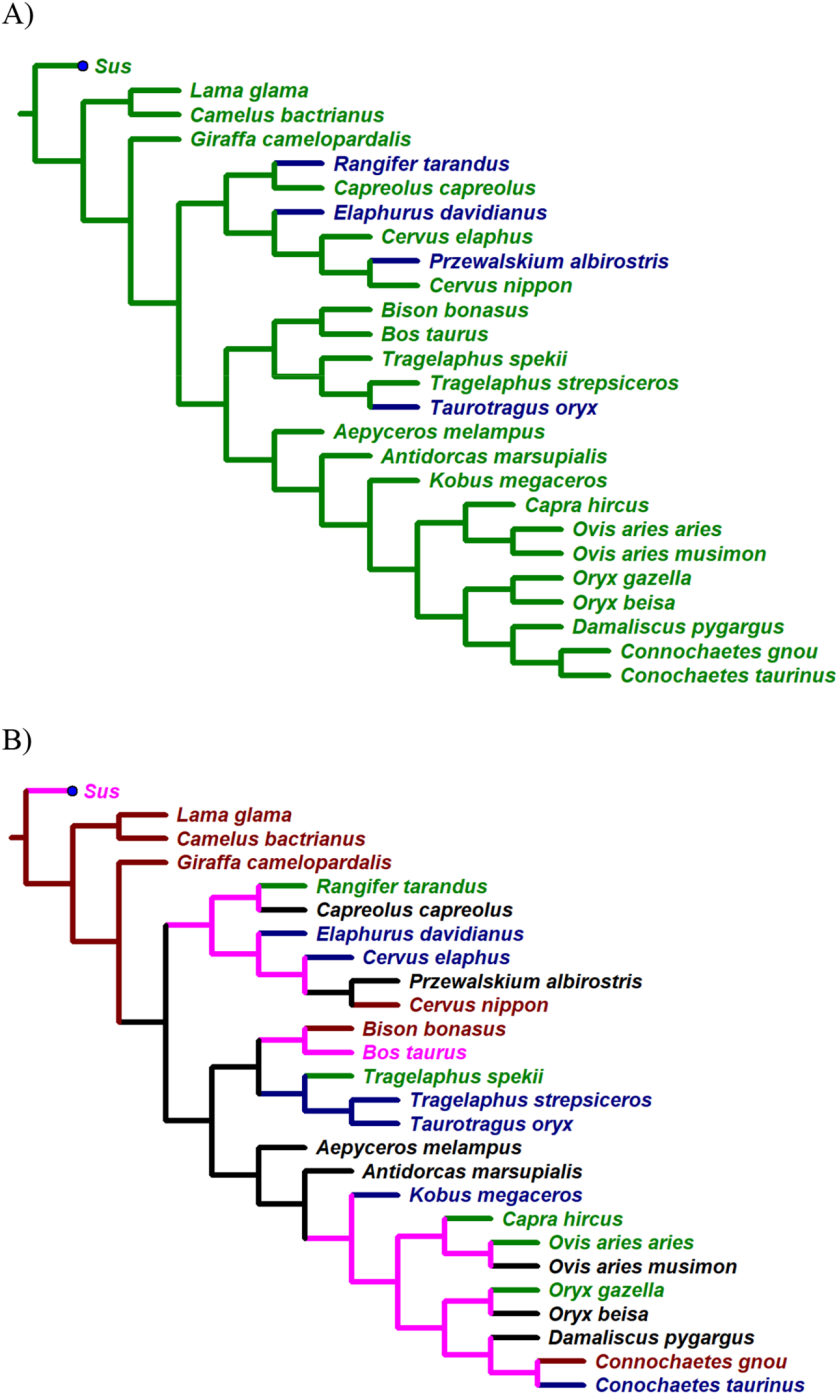
Evolution of knee-clicks (**A**) and OWMF length–width ratio (**B**) as reconstructed by the maximum parsimony approach. For more details see the body of text. Legend to (**A**): no knee-click in repertoire = green, knee-click present = blue. Legend to (**B**): type 1 of OWMF = green, type 2 = blue, type 3 = black, type 4 = brown, unresolved = pink.

However, these groups are not shared by all species within the same genus (*Cervus*, *Connochaetes*, *Oryx*, *Ovis*, *Tragelaphus*); only in two cases (both camelid species, and Greater kudu, and Common eland) is the particular shape shared by closely related species (i.e., in the sister-group configuration on the phylogenetic tree—Fig. 4B).

The discrimination between species included in this study is highly significant (Wilks’s Lambda = 0.0000036, F = 2063.9, p < 0.0001). Specifically, all specimens in 15 of the 20 species analysed were correctly classified using the classification matrix. Only several individuals were misclassified, specifically: two Impalas—once as an European roe deer and once as a White-lipped deer; two Springboks—once as an Impala and once as a White-lipped deer; two White-lipped Deer—once as an Impala and once as a Springbok; two European roe deer as Impala; and one Blue wildebeest as a Greater kudu.

All other statistical comparisons of the OWMF for body size, both digits, sex and age as main effects were non-significant (p-values > 0.05) indicating the conservative nature of the OWMF parameters. However, for age, the p-values were around 0.10 in all three inspected species (European roe deer, Domestic goat, and Domestic sheep), and for sex in the Giraffe and some bovids (Springbok, Black and Blue wildebeests, Blesbok, Giraffe, Gemsbok, European mouflon, Common eland, Greater kudu) with p-values around 0.08 or around 0.10 in the case of Impala.

The evolution of knee-clicks (Fig. 4A) was reconstructed successfully for all nodes of the phylogenetic tree. Interestingly, the evolution of OWMF, as reconstructed (Fig. 4B), was more complex, and OWMF types do not fit with the distribution of knee-clicks produced by some bovids and cervids.

## Discussion

**Figure 5:**
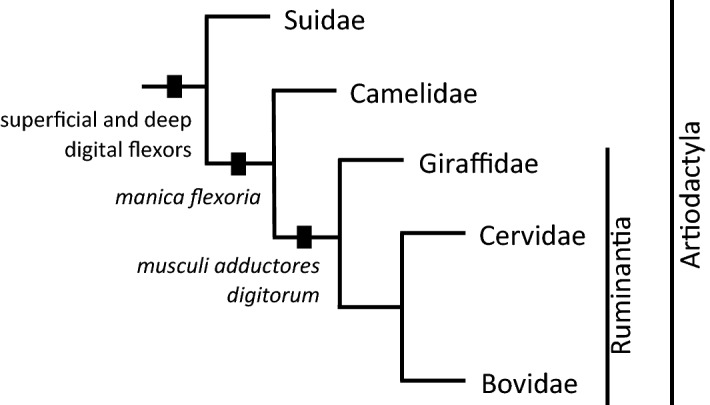
Simplified phylogenetic tree of even-toed ungulates (based on Hassanin et al.^44^) with a specification of the distribution of several morphological traits of the autopod mentioned in the “Discussion”.

The anatomy of the autopod of the forelimb is diverse in respect of specific phylogenetic clades of even-toed ungulates (for overview see Fig. 5). Specifically, the superficial and deep digital flexor are always on the forelimb of the even-toed ungulates, but *musculi adductores digitorum* can be found only in ruminant ungulates (Ruminantia). *Manica flexoria* is not formed in pigs as representatives of non-ruminant even-toed ungulates^22,35^. Constantinescu et al.^36^ dealt with the construction of the suspension apparatus of the spinal joint and the digital flexors on the forelimb of the llama (*Lama glama*). They confirmed the anatomical similarity of the muscles around the spinal joint of the llama and other ruminants. However, they found the absence of interflexor muscles (*musculi interfloxorii*) and the presence of *musculi lumbricales*, which are more typical of species with more than two digits^37,38^.

*Manica flexoria* arose independently in equids (Equidae)^39^, despite the lack of development of the *musculi adductores digitorum*^22,23,40^. The tendon of the superficial digital flexor forms a cuff with a strip of connective tissue that is located closer to the bone^30,41^. This band of connective tissue is histologically variable and is divided into membranous and tendinous types. The membranous type may take the form of a "synovial bridge", "fibrous bridge" or "broad synovial bridge". The tendinous type is then "symmetric X-crossing", "asymmetric X-crossing" or "oblique crossing"^42^. The surface digital flexor together with the auxiliary ligament band encircle the tendon of the deep digital flexor, thereby fixing it in position. Interestingly, *manica flexoria* is 2.5 cm longer on the horse's forelimb than on the pelvic limb^43^.

Besides phylogenetic inherence (see above), the observed conservatism of autopodium anatomy could be caused by functional constraints which optimize transport costs, escape and relaxing possibilities^13,14^. The same constraints seem to be valid for detailed parameters of the OWMF which exhibit marked similarities inside particular species. The similarity of the left and right side of the autopodia is expected^45,46^, whilst the distinctiveness between the OWMF of the third and fourth digits indicates some former selection for the symmetry of the autopodium which increases movement efficiency and endurance^25^.

On the other hand, the species-specific OWMF parameters indicate a differentiation, especially in the cervids and bovids, where the diversity has been higher than in giraffids or camelids (for former diversity see e.g., Janis^47^, for the current diversity see Groves and Grubb^48^). Since the recognized types based on the length–width ratio of the OWMF indicate very limited concordance to phylogenetic relationships (see phylogenetic trees in this study or for example, Hassanin et al.^44^), this might indicate a differentiation in some close relatives due to species-specific preferences for specific habitats or for preferred types of motion^21,49–51^. More in-depth taxonomic sampling would be beneficial to clarify the incidence, size, and shape diversity of the OWMF in Artiodactyla. The following taxa above the genus level (using Groves and Grubb^48^) are candidates for such further inspection: Tragulidae; Moschidae; Antilocapridae; Alceini and Muntiacini within Cervidae; and Boselaphini, Neotragini, Cephalophini and Oreotragini within Bovidae.

Animals of multiple ages were included in this investigation so as to study the possible dependence of the size and shape of OWMF on the age of the animal. The youngest studied were stillborn domestic cattle and domestic goats; the oldest was a 22-year-old European bison male. The shape and ratio of the length and width of the oval window were almost identical for all monitored categories within one species. Thus, it can be assumed that in older animals with increased cumulative mobility, there is no marked increase or decrease in the OWMF. It was also confirmed that animals that have never walked (stillborn due to foetal lung atelectasis) had an oval window developed on both attachment tendons of the adductors. This indicates that the OWMF is a physiological structure, and its presence is "programmed" in advance during intrauterine development.

The discovery of the non-described (not specified for example in Kolda^24^, Sisson and Grossman^35^, Najbrt et al.^22^, Nickel et al.^25^, Barone^38^, König and Liebich^23^, König et al.^52^, or in Böhmer et al.^53^ reporting on extensive mammal species sampled) oval window in the *manica flexoria* (OWMF) is surprising. A limited attention to some detailed configurations of the tendon apparatus in the autopodium, the limited conspicuousness of the OWMF, and using domestic cattle as the common model organism (e.g., Hedges)^54^ could be partly responsible for this oversight. This indicates that there are some gaps in basic comparative anatomical data (e.g. Guillerme and Cooper^55^, Conde et al.)^56^ and that some discoveries are still possible (e.g. Klima^57^, Crole and Soley^58^, Shadwick et al.^59^, Frey et al.^60^).

The appearance and function of the oval window in the OWMF is unclear. The most likely function is to help balance the pressure inside the ligament cuff and reduce the friction of the areas of the muscle tendons touching/rubbing against each other. However, this assumption has not yet been physically and technically confirmed and thus opens up a new field for further research. Another possible function is to increase the extent of digit flexion in species that have longer and wider OWMFs; *manica flexoria* with little or no oval window is more rigid and strong. This hypothesis is confirmed by the autopodies of the Guanaco and Bactrian camel with a large OWMF, which, compared to unguligrad even-toed ungulates, are semi-digitigrade with a higher range of movements in the toes^36^.

The importance of the OWMF for the self-flexion and extension of the digits of even-toed ungulates has not yet been elucidated, but there is importance for the veterinary medicine discipline; as example, it is possible to "pass" through the oval window in the *manica flexoria* inside the cuff with an arthroscope and to evaluate the damage and changes of the tendons, for example in septic tendosynovitis^61^. There is a sufficient distance between the bone base and the *manica flexoria* to allow the insertion of the endoscope and its movement in the individual muscle layers.

An interesting finding was that in domestic cattle, the OWMF does not develop on the forelimb even though it is developed in the hind limb (Supplementary Fig. S2). Its shape and the ratio of length and width are very similar to that of the European bison, which, however, has an oval window on both limbs. Perhaps this is due to the domestication of cattle, which, unlike its "wild" relatives, spends most of its life in a limited area of stables or pastures, the details surveyed in respect of these studies would be beneficial (e.g., O’Regan and Kitchener^62^, Keller et al.^63^, for references related to captivity-induced changes see also Robovský et al.)^64^.

The shape of the oval window in the *manica flexoria* among the inspected species was highly variable. The name "oval window" suggested and used is not entirely suitable for some species from this study. For example, in the reindeer the window shape is rather triangular with the base located proximally, whilst it is considerably elongated and spindle-shaped in the guanaco. However, more than three-quarters of the species in the study had an oval OWMF, thus this "functional" name was retained.

Another interesting finding was that the tendon of the deep digital flexor at the level of the OWMF is a few millimetres wider and higher than what it is proximal and distal to this site (Supplementary Fig. S1). In the histological assessment of this "swelling" in several selected individuals (Common eland, Bactrian camel, European bison), areas of cartilaginous tissue surrounded by normal connective tissue were found. The reason for this phenomenon is unclear, but it is most likely an adaptive response of the tendon to the mechanical demand due to the higher weight and age of the individual^65,66^.

The evolution of knee-clicks (Fig. 4A) was reconstructed successfully for all nodes of the phylogenetic tree. Knee-clicks seems to arise independently four times—specifically three times in cervids and once in bovids (in blue—Fig. 4A). However, the evolution of OWMF, as reconstructed (Fig. 4B), was more complex and not resolved for some nodes and clades in cervids and advanced bovids which prevents the description of the evolution of OWMF in detail. Since none of the absolute or relative (not shown) OWMF parameters fit exclusively with the distribution of knee-clicks produced by some bovids and cervids during movement, the mechanism responsible for this sound remain cryptic from the present anatomical perspective.

Therefore, the mechanism responsible for this specific sound requires further investigation and more analytical approaches. Knee-clicks have been documented in several species by acoustic analysis^3,8,9^, and Bro-Jørgensen and Dabelsteen^3^ identified knee-clicking as the honest signal of body size in the Common eland, using the comparison of acoustic parameters of knee-clicks and several other phenotype traits of inspected individuals. Despite the possibility to document knee-clicks readily by acoustic analysis^3,8,9^, it is not easily detectable from where this sound originates, as reported in our pilot study and as already reviewed by Mohr^5,11,67^ one century ago. Mohr^11,67^ mentioned several methods used by herself or other authors such as the fixing of particular limb regions by linen, experimental production of knee-clicks by bending of specific parts of limbs in dead individuals or using the stethoscope in live animals, but no progress has been done since that time. There are some obstacles in attempts to find the source of the knee-clicks’ sound. Firstly, it is necessary to use live animals or fresh carcass material before the development of rigor mortis, as documented by Mohr^11,67^ in the Reindeer, and as also shown by unsuccessful attempts to obtain knee-clicks post-mortem via different limb positions and pressure involved on different parts of the limbs in an attempt to mimic movement of the limb during walking within the present study. Secondly, since an acoustic camera could not identify a single area on the forelimb during sound emission on a walking eland antelope, it is necessary to use well cooperative/habituated^11^ representatives of species producing this type of sound in vivo, moreover under standardized conditions in order to minimize sources of the (acoustic) noise under natural or captive conditions. Finally, any generalization obtained from one species might be limited due to independent origins of knee-clicks in even-toed ungulates, as detected in the present study.

## Material and methods

To find the source of the emitted clicking sound on a tame adult eland bull kept at Eland farm (Czech University of Life Sciences Prague), an acoustic camera (Norsonic AS, Tranby, Norway) provided and operated by Ekola Ltd. Prague was used. The sound(s) were measured in a barn, where the eland herd is housed, from a distance of 3–4 m, during the walking of the inspected individual, when the clicking sound was clearly emitted. As an additional trial, the whole forelimb of another adult eland bull was evaluated post-mortem (this bull was slaughtered due to regular reduction as part of farmed herd management). Before slaughter, this animal was emitting clicking sounds during walking. The whole skin-on forelimb was removed 1.5 h after post-mortem and stored at 7 °C until the next day, when the forelimb was transported to the biomechanical lab at the Department of Anatomy and Biomechanics, (Faculty of Physical Education and Sport Regulations, Charles University Prague). A stethoscope, sound recorder and palpation were used for sound and vibration detection in endeavours to obtain knee-clicks post-mortem via different limb positions and pressure involved on different parts of the limbs in an attempt to mimic movement of the limb during walking.

To conduct a comparative study of thoracic autopodia (manus—from wrist to hoof), 25 species of even-toed ungulates (Artiodactyla, see Asher and Helgen, and Prothero et al.)^68,69^ of four families (Camelidae, Giraffidae, Cervidae and Bovidae), consisting of various ages and sexes were investigated (for detail list see Table 2) (it contains references ^49,70–83^).

**Table 2:** List of taxa (ordered alphabetically according to the scientific name) with common names, average weight of species extracted from the literature, and individuals inspected in this study.

Scientific name	Common name	Avg. weight (kg)	Avg. weight (kg)	Avg. weight (kg)	Sources	N	Sex	Age
		M	F	M + F				
*Aepyceros melampus*	Impala	56.9	43.8	50.35	^70^	15	13M/2F	15A
*Antidorcas marsupialis*	Springbok	40.7	35.5	38.1	^70^	18	8M/10F	18A
*Bison bonasus*	European bison	718	423	570.5	^70^	1	1M	1S
*Bos taurus taurus*	Domestic cattle	384	327.5	355.75	^70^	6	3M/3F	2J + 2A + 2S
*Camelus bactrianus bactrianus*	Bactrian camel	600	450	525	^71,72^	2	2F	2S
*Capra hircus hircus*	Domestic goat	50	30	40	^73^	6	3M/3F	2J + 2A + 2S
*Capreolus capreolus*	European roe deer	24.2	23.4	23.8	^70^	6	3M/3F	2J + 4A
*Cervus elaphus*	Red deer	185.1	140.2	162.65	^70^	3	1M/2F	2A + 1S
*Cervus nippon pseudaxis*	Indochinese sika deer	90	50	70	^74^	4	2M/2F	4A
*Connochaetes gnou*	Black wildebeest	166.7	135	150.85	^70^	6	3M/3F	6A
*Connochaetes taurinus taurinus*	Blue wildebeest	235.3	184.9	210.1	^70^	10	5M/5F	10A
*Damaliscus pygargus phillipsi*	Blesbok	70	60	65	^75^	8	4M/4F	8A
*Elaphurus davidianus*	Père David's deer	207.3	149.9	178.6	^70^	4	2M/2F	4A
Giraffa camelopardalis*	Giraffe*	1190.2	814.3	1002.25	^70,76–78^	16	8M/8F	15A + 1S
*Kobus megaceros*	Nile lechwe	105	75	90	^49,75,79^	1	1M	1A
*Lama glama guanicoe*	Guanaco	109.5	99	104.25	^70,71^	1	1M	1S
*Oryx beisa beisa*	Beisa oryx	176.4	161.7	169.05	^80^	4	2M/2F	2A + 2S
*Oryx gazella*	Gemsbok	178	166.4	172.2	^70^	12	6M/6F	12A
*Ovis aries aries*	German gray heath sheep	67.9	44.9	56.4	^70^	6	3M/3F	2J + 2A + 2S
*Ovis aries musimon*	European mouflon	42.5	35	38.75	^81^	6	2M/4F	6A
*Przewalskium albirostris*	White-lipped deer	204.2	125	164.6	^82^	4	2M/2F	2A + 2S
*Rangifer tarandus*	Reindeer	145	85.8	115.4	^70^	3	1M/2F	1A + 2S
*Taurotragus oryx*	Common eland	647.3	415.8	531.55	^70^	6	3M/3F	6A
*Tragelaphus spekii gratus*	Western sitatunga	115	55	85	^83^	1	1M	1S
*Tragelaphus strepsiceros*	Greater kudu	240.8	159.2	200	^70^	7	4M/3F	7A
Total						156	82M/74F	8J + 129A + 19S

*We evaluated a population equivalent to the Angolan giraffe (= *angolensis* Lydekker, 1903).

*avg.* average, *N* sample size, sex: *F* female, *M* male, *age*: *A* adult, *J* neonate or juvenile, *S* senescent.

Altogether 312 autopods of the forelimb originating from 156 individuals were studied (Table 1). The origin of the animals varied and included zoos in the Czech Republic (Brno Zoo, Dvůr Králové Zoo, Chomutov Zoo, Jihlava Zoo, Olomouc Zoo, Ostrava Zoo, Pilsen Zoo, Prague Zoo, and Ústí nad Labem Zoo), as well as private breeders, associations, and school institutions (VFU Brno, CZU Prague, Miskovice u Kutné Hory). Additional material was also obtained during the harvest of game species in the Republic of South Africa (Game farms near Bredasdorp and Witsand—Springbok, Black wildebeest and Blesbok; Game ranches close to Modimole—Impala, Blesbok and Blue wildebeest) and game ranches in Namibia near Windhoek and Kalkfeld (Springbok, Giraffe and Gemsbok). In this case, these were wild animals kept under minimal husbandry conditions intended for meat production. As these animals were all from either cadavers (who had died from various causes) or were collected from carcasses that were part of a standard harvesting for meat management routine, no animal ethics approvals were required. The sex of the animals used in the study was almost evenly represented with a slight predominance of males over females (82 males and 74 females). Only two age groups were distinguished in the study. The first and most numerous were sexually and physically mature individuals (148 individuals), followed by new-borns and calves (8 individuals; hereafter labelled as non-adult individuals).

Each autopodium underwent a thorough anatomical autopsy focusing on the macroscopic structure of superficial and deep digital flexors and short digital muscles, and their tendons, as well as the topographic relationships of the structures around the *manica flexoria* and sesamoid bones. First, the skin was removed from the autopods and then the *manica flexoria* was dissected from the bone base (Supplementary Fig. S3). The presence or absence of any different structure from known anatomical parts or distinct species, sexes and age groups were determined. A yet to be described opening, a so-called "oval window" of the *manica flexoria* (abbreviated as OWMF) (Supplementary Fig. S4) was observed in the adduct tendon on the tendon’s surface facing the bone; its shape and bilateral symmetry were determined. Using a calliper or a metric band, two dimensions of this hole were obtained, namely its length and width (Supplementary Fig. S5). The term length is defined as the dimension between the most proximal and the most distal edge of the window, the width the distance between the axial and abaxial edge. Autopsies were performed at the Institute of Anatomy, Histology and Embryology (Veterinary University in Brno) and in laboratories designated for that purpose at Czech University of Life Sciences Prague and Jiří Orten Grammar School in Kutná Hora. The material originating from individuals obtained by controlled harvesting in South Africa and Namibia had to be subjected to on-site autopsy in field conditions and in local abattoirs.

The obtained measurements (length and width of the OWMF) are specified in millimetres (mm) as means ± SD (Table 2) but were analysed statistically as primary values in respect of sex (male vs female), age (non-adult vs adult), body side (left vs right), digit (third vs fourth digit) and species (all species included in this study). Although the Shapiro-Wilks test recognized some data as not being deviant from normality, the same nonparametric test variant (Mann–Whitney test) for all species was applied so as to minimise the risk of false positive results (type I error) due to small sample sizes. Since non-adult individuals were available for only three species (Domestic goat*,* European roe deer and Domestic sheep), the effect of age was only evaluated in these species, whilst for species comparisons, only adult specimens were included to maximize comparability. In summary, species differences were analysed using Discriminant analysis, and other category differences using the non-parametric Mann–Whitney test and Sign test. Significance was considered when p ≤ 0.05. Microsoft Excel under Microsoft 365 and Statistica ver. 13.5.0.17 (copyright TIBCO Software) were used for calculations and statistical comparisons. Some species were not analysed within all statistical comparisons due to their sample size being less than three individuals (Table 1).

To compare the evolutionary distribution of knee-clicks and OWMF, the absolute and relative size of the OWMF (see Table 2) using body weight as a proxy of the body size, were utilised. Sources of body weights^49,70–83^ are specified in Table 2. The evolution of knee-clicks and OWMF (specifically, the length–width ratio) was optimized by NONA (ver. 2.0) and WINCLADA interface (ver. 1.00.08^84^) using the unweighted maximum-parsimony approach on a simplified and consensual phylogenetic tree adopted from Pitra et al.^85^, Hernández Fernández & Vrba^86^, Hassanin et al.^44^ and Chen et al.^87^. The topology of the phylogenetic tree was constrained for reconstruction. No preference to ACCTRAN nor DELTRAN optimization were given when alternative reconstructions were of equal cost.

### Graphics

Photographs used were taken by M. P. Phylogenetic trees were produced using WINCLADA (v1.00.08^84^), IrfanView v4.57—64 bit downloaded from https://www.irfanview.com/ and Microsoft 365 interface downloaded from https://www.microsoft.com/cs-cz/microsoft-365.

### Ethical statement

For the study, material was received from animals slaughtered for meat production, or euthanised due to health reasons or that had died naturally. No single animal was slaughterer or euthanised to gather material/tissue for this study and all causes of death were unrelated to the musculoskeletal system. All procedures followed Czech or international laws for manipulation and culling of farmed animals or veterinary and husbandry laws applied to zoo gardens. No extra permission/ethical clearance or approval by an ethical committee was necessary since all manipulations with study material and procedures were done post-mortem and not required by Czech legislation.

## Supplementary Information

Supplementary Information.

## Data Availability

The data matrix is available in the Supplementary online material, other data subsets used and/or analysed during this study are available from the corresponding author on request.
