# Supplementary material for: Newly described anatomical opening on forelimb tendon in the artiodactyls and its relation to knee clicks
Source: Sci Rep. 2022 Mar 14;12:4362. doi: 10.1038/s41598-022-08303-z (PMC8921199; doi:10.1038/s41598-022-08303-z)
Supplement: Supplementary file 1 — Supplementary Information. [file 41598_2022_8303_MOESM1_ESM.docx]

Supplementary material

**Content**

**Fig. S1.** Superficial and deep digital flexors in Guanaco (A), and Bactrian camel after the “replacement” of underdeveloped *musculi adductores digitorum* has been dissected (B).

**Fig. S2.** Presence and absence of the “oval window” in *manica flexoria* of the pectoral limb. A – Domestic cattle – hind limb, B – Domestic cattle – pectoral limb, C – European bison – pectoral limb.

**Fig. S3.** Muscles on the palmar surface of the pectoral limb of Common eland.

**Fig. S4.** The oval window in the *manica flexoria* (view of the adductor surface). A – Père David's deer, B – European roe deer, C – Guanaco.

**Fig. S5.** Measuring the length and width of the oval window – in a Beisa oryx. Photos by M. P.

**Appendix S1.** Additional morphological comments.

**Appendix S2.** References used in Supplementary material.


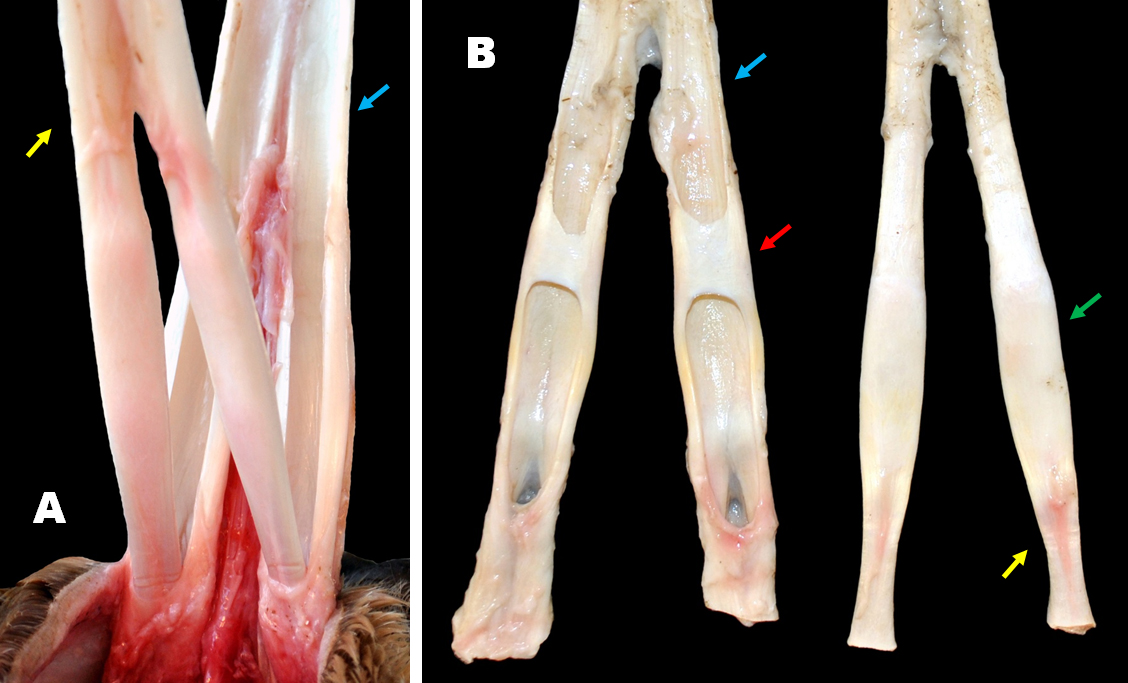


**Figure S1. Superficial** (the blue arrow) **and deep digital flexors** (the yellow arrow) **in Guanaco (A), and digital flexors in Bactrian camel after the “replacement” of underdeveloped *musculi adductores digitorum* has been dissected (B)**: the view of the area facing the bone (the blue arrow), the place of the reinforced digital flexor tendon in the OWMF level (the green arrow), the fibrous “bridge” formed by connecting the axial and abaxial edge of the superficial digital flexor (the red arrow). Photos by M. P.


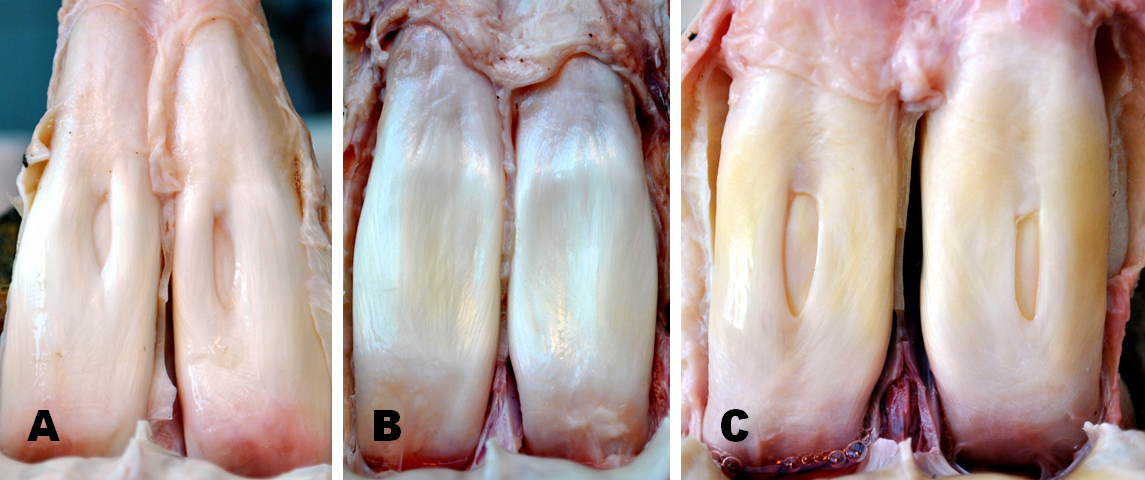


**Figure S2. Presence and absence of the “oval window” in *manica flexoria* of the pectoral limb. A – Domestic cattle – hind limb, B – Domestic cattle – pectoral limb, C – European bison – pectoral limb.** Photos by M. P.


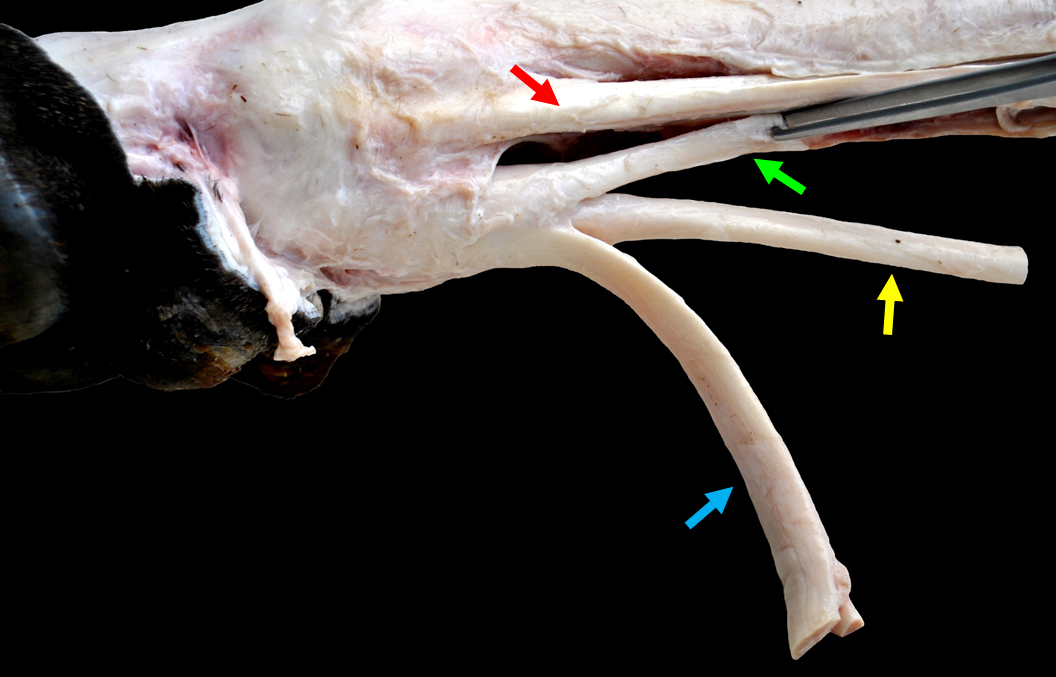


**Figure S3. Muscles on the palmar surface of the pectoral limb of Common eland** (lateral view): *musculus. flexor digitorum superficialis* (the blue arrow), *musculus flexor digitorum profundus* (yellow arrow), *musculi adductores digitorum* (the green arrow) and *musculi interossei* (the red arrow). Photos by M. P.


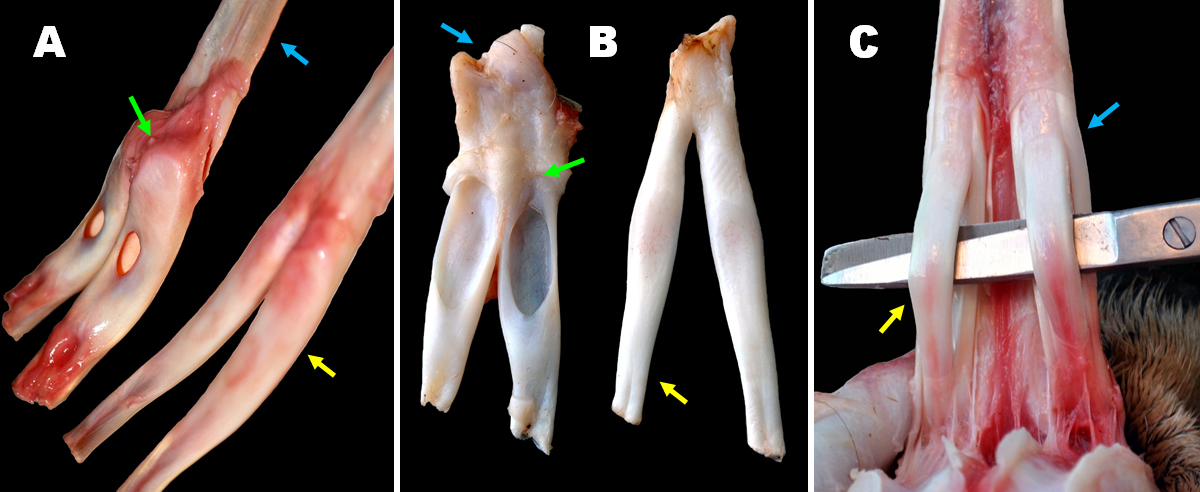
**Figure S4. The oval window in the *manica flexoria* (view of the adductor surface). A – Père David's deer, B – European roe deer, C – Guanaco.** *Musculus flexor digitorum superficialis* (the blue arrow), *musculus flexor digitorum profundus* (the yellow arrow), *musculi adductores digitorum* (the green arrow). Photos by M. P.


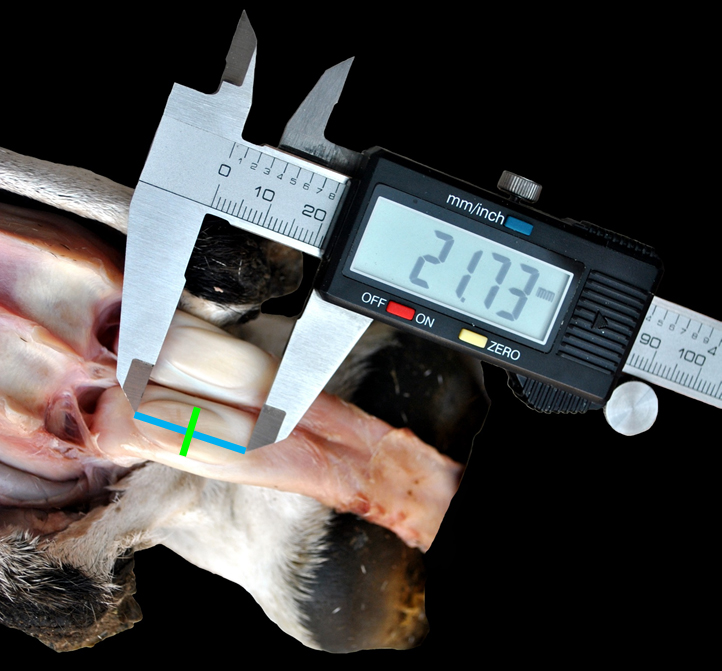
**Figure S5. Measuring the length (blue) and width (green) of the oval window (view of the adductor surface) – in a Beisa oryx.** Photos by M. P.

**Appendix S1. Additional morphological comments.**

*Musculus flexor digitorum superficialis* recedes into the *epicondylus medialis humeri* and is divided into two components in even-toed ungulates. The surface component is flattened on the sides and about three times more powerful than the deep component. Both components pass just above the carp into tendons, which are separated from each other by the *retinaculum flexorum* (Kolda 1950; Sisson & Grossman, 1953). The tendon of the surface component is located externally, while the tendon of the deep component is located together with the deep digital flexor in the *canalis carpi*. At about half the length of the axes of metacarpals III and IV, the tendons of the two components of the surface flexor join. It thus forms a short joint tendon, which, however, splits again into two arms above the basic joint of the digit (*articulatio metacarpophalangea*) (Najbrt et al. 1980, Barone 2000). Each of the arms (for the third and fourth digits), with the *adductor digitorum* on its side, forms a fibrous tube (cuff), through which the attachment tendon of the deep digital flexor runs. Distal to it, the two arms split into three attachment branches. The lateral branches are weaker and clamp to the *torus phalangis mediae*, the massive middle branch clamps between them (Ellenberger & Baum 1977, König & Liebich 2003).

Compared to the previous muscle, the *Musculus flexor digitorum profundus* consists of three heads; the humeral caput, radial caput and ulnare caput. The arm head of the deep digital flexor is the most massive and recedes from the *epicondylus medialis humeri*. The spindle and elbow head are from the proximal ends of the respective bones (Koch 1960, Najbrt et al. 1980). The tendons of all three heads merge at the wrist level into a single common tendon of the deep digital flexor that passes in the *canalis carpi* (Kolda 1950, Nickel et al. 1986) and splits into two branches (for the third and fourth digits) in the distal third of the metacarpus. At the level of the foam joint, these branches penetrate the fibrous tubes formed by the connection of the surface digital flexor and the digital adductors. After the deep digital flexor tendons have exited the *manica flexoria*, they are clamped to the *tuberculum flexorium* of the distal digit joint (Nickel et al. 1986, Barone 2000).

*Musculi adductores digitorum*, namely, *adductor digiti II* and *adductor digiti V* are two short digital muscles that are completely fibrous in cattle (Najbrt et al. 1980). In small ruminants, especially goats and young individuals, the proximal end of the muscle is still permeated with muscle fibers, even though the muscle is still more than two-thirds fibrous. Both digital adductors start together with the *musculi interossei (musculus interosseus III et IV)* on the palmar surface of the base of the metacarpus (König & Liebich 2003). About halfway up the length of the metapodia, they separate from the bone base and lead to the palmar surface. At the edge of the spinal joint of the digit the two adductors connect to the corresponding tendon of the superficial digital flexor, with which they clamp to the *torus palmaris phalangis mediae* (Najbrt et al. 1980; König & Liebich 2003). A large number of anatomical publications (especially older print dates) indicate that *musculi adductores digitorum* form a surface component of the interosseus medius muscle (Kolda 1950, Koch 1960, Nickel et al. 1986, Waibl et al. 2001) or function as its *ligamentum acessorium* (Sisson & Grossman, 1953; Bertagnoli et al. 2012). From the embryological point of view, however, these are two separate muscles that have only a common distance on the palmar surface of the base of the metacarpal bone. The *manica flexoria* therefore includes *musculi adductores* and not *musculi interossei* (Ellenberger & Baum 1977, Najbrt et al. 1980, König & Liebich 2003, I.C.V.G.A.N. - NAV 2012).

**Appendix S2. References used in Supplementary material (in alphabetical order).**

| Barone, R. *Anatomie Comparée des Mammiferes Domestiques. Tome 2, Arthrologie et myologie* (Vigot, 2000).  Bertagnoli, A., Raber, M., Morandi, N., Mortellaro, C. M. & Steiner, A. Tenovaginoscopic approach to the common digital flexor tendon sheath of adult cattle: Technique, normal findings and preliminary results in four clinical cases. *Vet. J.* **191**, 121–127. <https://doi.org/10.1016/j.tvjl.2010.12.009> (2012). |
| --- |
| I. C. V. G. A. N. *Anatomica Veterinaria*, Fifth Edition (Editorial Committee of I. C. V. G. A. N., 2012).  Koch, T. *Lehrbuch der Veterinär - Anatomie. Band I, Bewegungsapparat* (VEB Gustav Fischer Verlag Jena, 1960). |
| Kolda, J. *Comparative Anatomy of Domestic Animals III.-IV. Arthrology, Myology Including the Mechanics of Movement* (Studentská organizace čs. veterinárních mediků, 1950) (in Czech).  König, H. E. & Liebich, H. G. *Anatomy of Domestic Mammals. Part 1: Musculoskeletal System* (H&H, 2003) (in Czech).  Najbrt, R. *et al.* *Veterinary Anatomy 1* (SZN, 1980) (in Czech).  Nickel, R., Schummer, A. & Seiferle, E. *The Anatomy of the Domestic Animals, Volume 1, The Locomotor System of the Domestic Mammals* (Verlag Paul Parey, 1986).  Waibl, H., Herrmann, J., Rehage, J., Lorenzi, P. & Constantinescu, G. Applied anatomy of the distal “vinculum tendinis” in the fetlock tendon sheath of the hindlimb in cattle. *Dtsch. Tierarztl. Wochenschr.* **108**(6), 261–263. |
